# Safety of lotilaner flavoured chewable tablets (Credelio^TM^) after oral administration in cats

**DOI:** 10.1186/s13071-018-2969-3

**Published:** 2018-07-13

**Authors:** Emmanuelle A. Kuntz, Srinivas Kammanadiminti

**Affiliations:** 1 Elanco Animal Health, Mattenstrasse 24a, CH-4058 Basel, Switzerland; 2 Elanco Animal Health, 2500 Innovation Way, Greenfield, IN 46140 USA

**Keywords:** Lotilaner, Credelio™, Safety, Cat, Oral

## Abstract

**Background:**

Lotilaner is a new member of the isoxazoline class for treatment of flea and tick infestations in cats. This laboratory study with lotilaner vanilla-yeast flavoured chewable tablets (Credelio^TM^, Elanco) investigated the safety in healthy kittens starting at 8 weeks of age in a randomized, blinded, parallel-group design. Lotilaner tablets were given orally once a month over eight months at one, three and five times the upper level of the maximum recommended dose range (26 mg/kg).

**Methods:**

The safety of lotilaner flavoured chewable tablets was assessed in healthy kittens when administered orally every 4 weeks for 8 months at the highest recommended dose rates, i.e. 1× (26 mg/kg) and at elevated dose rates, i.e. 3× (78 mg/kg) and 5× (130 mg/kg). Sixteen male and 16 female healthy 8-week-old kittens, with a mean body weight of 0.79 kg and 0.75 kg, respectively, were randomized to an untreated control group or lotilaner groups at dose rates of 26 mg/kg (1×), 78 mg/kg (3×), or 130 mg/kg (5×) every four weeks over eight months. The control group was sham-dosed. All animals were fed within 30 minutes prior to treatment. Safety assessment included general health observations, detailed clinical observations, complete physical/neurological examinations, including ophthalmological examinations, electrocardiographic (ECG) and clinical pathology evaluations (haematology, clinical chemistry and urinalysis), food and water consumption, body weight, pharmacokinetic blood collections, organ macroscopic and microscopic examinations.

**Results:**

Systemic exposure to lotilaner was confirmed during the course of the study in all treated animals with the exception of the control group. No treatment-related effects were seen on daily clinical observations, food consumption (wet), ophthalmoscopic, physical/neurological and microscopic examinations. Statistically significant differences were recorded in some of the clinical pathology parameters, body weights, food consumption (dry), electrocardiograms, and organ weights, but none of the recorded observations was considered to be of clinical relevance.

**Conclusions:**

Lotilaner, when administered once monthly over eight months at the highest recommended dose and overdoses of three- and five-fold, to 8-week-old healthy kittens, is well tolerated.

**Electronic supplementary material:**

The online version of this article (10.1186/s13071-018-2969-3) contains supplementary material, which is available to authorized users.

## Background

Infestation by ticks and fleas is a significant health problem in cats and dogs as these ectoparasites act as vectors for several feline or canine zoonotic diseases. Hence, controlling ticks and fleas is an important part of health management of pets [1, 2]. While several products are available for flea control, options for tick control are limited in cats. In this context, having one product that treats both ticks and fleas is desirable.

Isoxazolines are a new chemical class of synthetic parasiticides demonstrated to be effective against ticks and fleas [3]. Isoxazolines are safe for mammals due to their non-competitive antagonism to GABA (gamma-aminobutyric acid) receptor, with higher selectivity for GABA receptors in insects or ticks, than for those in mammals, including humans. They bind to chloride channels in nerve and muscle cells, which blocks the transmission of neuronal signals [4–6].

Lotilaner is a novel isoxazoline recently approved as Credelio™ in dogs. Laboratory and field studies have established lotilaner as a valuable drug in the management of tick and flea infestations in dogs [7–14]. The drug has a half-life of approximately four weeks in cats thereby assuring adequate blood levels for at least one month [15]. Efficacy evaluation of lotilaner was also conducted in cats for the same indication [16–18]. Prior to approval of the product, its safety must be established in the target species.

The objective of this study was to evaluate the safety of lotilaner flavoured chewable tablets, formulated and developed specifically for cats, after repeated oral administration at the maximum recommended dose and multiple overdoses in eight-week-old kittens. The treated groups received lotilaner once a month over 8 months. The recommended (minimum) monthly dose rate of lotilaner is 6 mg/kg with a weight band of four resulting in a maximum recommended dose rate of 26 mg/kg.

## Methods

### Regulatory compliance

This randomized, controlled, blinded study was conducted with reference to the guidelines for evaluating the target animal safety of new pharmaceuticals (VICH Guideline 43), and to recognized quality assurance standards (United States Food and Drug Administration (FDA) Good Laboratory Practice (GLP) Regulations, 21 Code of Federal Regulations (CFR) Part 58 and the Organization for Economic Cooperation and Development (OECD) Series on Principles of Good Laboratory Practice and Compliance Monitoring, Number 13)] [19–21]. This manuscript was prepared in compliance with the ARRIVE Guidelines Checklist for animal *in vivo* experiments [22].

### Animal management

A total of 20 male and 20 female experimentally naïve domestic short hair kittens approximately 6 weeks of age at receipt were acclimatized to the controlled environment for two weeks prior to treatment start to facilitate baseline data collection. Sixteen male and 16 female animals (weighing 0.62–0.98 kg and 0.66–0.91 kg, respectively, at randomization) were selected and assigned to the control or treatment groups on study day -1. Upon arrival, the cats were group-housed (four/cage, same sex); on study day -1 and for the whole study period, the cats were individually housed in stainless steel mobile cages with plastic-coated mesh flooring containing animal enrichment. At arrival and through 10 weeks of age, the animals were offered daily 60 to 80 g of canned food (Purina Dietetic Management (DM), St-Louis, USA) and dry food (Lab Diet® Certified Feline Diet #5003, PMI Nutrition International, Inc., St-Louis, USA) while after 10 weeks, only dry food was offered *ad libitum* to all animals with the exception of limited periods prior to treatment administration. Tap water was available *ad libitum* to all animals. On administration days, the cats were fasted for 6 hours prior to the first dosing which increased by an hour for each subsequent monthly dosing until 12 h of fasting was reached by the 6th dose. Fasting duration of a maximum of 12 h was continued subsequently. On treatment days, all animals were offered 60 to 80 g (1st through 5th doses) or 120 to 160 g (6th through 8th dose) of canned food (Purina DM) along with the daily ration of dry Lab Diet® 30 min prior to dosing to improve bioavailability [15].

### Randomisation, blinding and treatment

Sixteen male and 16 female animals were randomly allocated on study day -1 to the control group or to the treatment groups based on homogenous distribution of body weight and sex (4 males and 4 females per group) (Table 1). The four groups were: Group 1: untreated control (sham-dosed with 3 ml of tap water); Group 2: lotilaner flavoured chewable tablets at a target dose level of 26 mg/kg (1×); Group 3: lotilaner flavoured chewable tablets at a target dose level of 78 mg/kg (3×); Group 4: lotilaner flavoured chewable tablets at a target dose level of 130 mg/kg (5×).

**Table 1:** Range of lotilaner dose rates administered to each of the study groups

	Day of dosing							
	1	29	57	85	113	141	169	197
26 mg/kg (1×)								
Male	27.0–33.8	26.1–31.0	23.5–25.9	25.4–27.8	24.6–27.8	25.0–27.3	25.1–27.4	25.1–26.2
Female	27.9–32.0	25.0–25.5	24.7–26.1	24.8–27.0	24.3–27.8	25.8–28.1	25.6–28.0	24.7–27.9
78 mg/kg (3×)								
Male	71.6–87.0	78.8–84.5	80.0–81.2	77.4–80.9	78.5–81.5	77.3–79.7	78.8–80.8	77.1–78.3
Female	76.9–84.5	76.8–84.5	77.6–79.6	76.7–80.3	77.9–81.1	76.5–79.2	78.2–80.0	76.8–79.7
130 mg/kg (5×)								
Male	123.5–135.5	130.1–137.4	130.8–134.0	127.9–130.3	131.8–132.6	129.2–131.7	128.8–134.0	128.8–129.8
Female	121.7–135.2	132.3–136.4	133.3–135.7	127.4–132.1	128.9–132.9	128.9–132.5	131.4–133.3	129.5–131.9

All personnel were unaware of the treatment group allocations except those involved in administration of treatments. Histopathological evaluation was conducted unblinded.

### Test article administration

The target dose levels were selected based on the minimum therapeutic dose (6 mg/kg), when applying a dose banding for the available tablet strengths of four times the target dose rate leading to approximately the maximum therapeutic dose of 26 mg/kg and the requirement to test multiples of the maximum therapeutic dose, i.e. 3× and 5× doses. Doses for each individual animal were based on each body weight measured the day prior to each day of dosing. Tablets (commercial tablets size, not scored) were provided, with lotilaner amounts of 12 or 48 mg. An appropriate combination of the smallest, i.e. 12 mg or the largest, i.e. 48 mg tablets was administered to achieve as close as possible to the individual target dose. As lotilaner absorption has been shown to be increased by food, cats were fed within 30 min prior to each dosing [15].

Tablets were administered *per os* once every four weeks for eight months (on study days 1, 29, 57, 85, 113, 141, 169 and 197). Three milliliters of water was then given after each administration and the mouth checked to ensure the tablets had been swallowed. The control animals were sham-treated with 3 ml of tap water.

### General health observations

All animals were observed twice daily approximately 6 h apart for morbidity, mortality and injury.

### Detailed clinical observations, electrocardiographic and ophthalmoscopic examinations

A detailed clinical examination of each cat was performed on study days -13, -2 and -1 and then on each dosing day 8 h (± 1 h) post-dose, once weekly thereafter, and on the day of study completion (study day 225). Observations included, but were not limited to, evaluation of skin, fur, eyes, ears, nose, oral cavity, thorax, abdomen, external genitalia, limbs and feet, respiratory and circulatory signs, autonomic effects such as salivation, and nervous system effects including tremors, convulsions, reactivity to handling, and unusual behavior.

Electrocardiographic (ECG) recordings were performed while the cats were under sedation with ketamine and acepromazine [0.1 ml/kg body weight of ketamine (100 mg/ml) and 0.1 ml of acepromazine (10 mg/ml)] during the acclimation phase on study days -6, and 59, 143, 199 and 224. The ECG tracings from each animal were examined by a certified veterinary cardiologist for the following variables: heart rate, RR interval, PR interval, QT intervals and QRS duration.

Ophthalmoscopic examinations were conducted by a board certified ophthalmologist on all animals on study days -4, 99 and 211.

### Body weights and food consumption

Body weights for all animals were recorded during the acclimation phase (study days -12, -9, -7, -5, -2 and -1) and at least once weekly during the study. Food consumption (dry and wet food) was measured and recorded daily.

### Physical/neurological examinations

Complete physical and neurological examinations were conducted on study days -6, 7, 35, 63, 91, 119, 147, 175, 203 and 224. Assessments of toxicity and health included general condition and behavior; general ocular without ophthalmoscope; integument; musculoskeletal; gastrointestinal; body temperature; cardiovascular and respiratory including assessment by auscultation; reproductive system; lymphatic, urinary and nervous systems. The neurological assessment included observation for nystagmus, pupillary response, extensor thrust (muscle tone), righting reflex, startle reflex, proprioception, and locomotor activity.

### Clinical pathology and urinalysis

Blood samples were collected from each animal from the jugular vein for the evaluation of hematology, clinical chemistry and coagulation variables at pre-test (study day -8 for hematology and clinical chemistry; study day -5 for urinalysis and study day -1 for coagulation), and on study days 8, 28, 36, 56, 64, 84, 92, 112, 120, 140, 148, 168, 176, 196, 204 and 223. Urine samples were collected (prior to study day -4, 7 days after the first dose; then prior to and 7 days after each subsequent dose, and prior to termination) by replacing the litter in the litter box with NoSorb® for at least 16 h. The hematology profile included the following parameters: leukocyte count (total and absolute differential), erythrocyte count, hemoglobin, hematocrit, mean corpuscular hemoglobin, mean corpuscular volume, mean corpuscular hemoglobin concentration (calculated), reticulocytes (aggregate and punctuate) and platelet count. The coagulation parameters included prothrombin time, activated partial thromboplastin time (APTT), and fibrinogen. The clinical chemistry profile included alkaline phosphatase, total bilirubin (with direct bilirubin if total bilirubin exceeded 1 mg/dl), aspartate aminotransferase, alanine aminotransferase, gamma glutamyl transferase, urea nitrogen, creatinine, total protein, albumin, globulin and albumin/globulin ratio (calculated), glucose, total cholesterol, triglycerides, electrolytes (sodium, potassium, chloride), calcium and phosphorus. Urinalysis included the determination of volume, color and appearance, specific gravity, pH, protein, glucose, bilirubin, ketones, blood, leukocytes, urobilinogen and microscopy of centrifuged sediment.

### Whole blood and pharmacokinetic analysis

For pharmacokinetic determination, blood samples were collected from all animals *via* the jugular vein for the determination of whole blood concentrations of lotilaner at predose (study day -1, 29, 57, 85, 113, 141, 169 and 197); 4 h post-dose on study day 1; predose and 4 h post-dose on study day 113; 24 h post-dose on study days 2, 30, 58, 86, 114, 142, 170 and 198; and on study days 4, 8, 15, 22, 116, 120, 127, 134, 200, 204, 211, 218 and 225. The samples were analyzed for determination of lotilaner concentrations using a validated method by HPLC-MS/MS [15]. The pharmacokinetic parameters were calculated from the individual concentration *vs* time profiles *via* non-compartmental analysis. The pharmacokinetic parameters included peak values (C_max_), terminal half-life (T_1/2_), area under the curve (AUC), and accumulation ratio.

### Gross and microscopic evaluations

At study termination (study day 225), the cats were humanely euthanized by an intravenous injection of sodium pentobarbital solution followed by exsanguination. Gross examinations of tissues were carried out on all animals according to VICH GL 43 under the supervision of a veterinary pathologist [19]. Microscopic examinations were done by a board certified veterinary pathologist.

### Statistical methods

All statistical analyses were performed using software package SAS/STAT® (Version 14.1, Version 9.4 of the SAS System for Windows, Copyright© 2002-2012 by SAS Institute Inc., Cary, NC, USA).

Endpoints measured once post-treatment (organ weights and pharmacokinetic parameters) that did not include a pre-treatment measurement were analyzed using analysis of variance (ANOVA) with ‘treatment’, ‘sex’, and ‘treatment by sex’ as fixed effects [23]. Endpoints measured multiple times post-treatment that included a pre-treatment measurement (body weights, ECG parameters, clinical chemistry, hematology, urinalysis, coagulation, dry food consumption, and wet food consumption) were analyzed using repeated measures analysis of covariance (RMANCOVA) with ‘treatment’, ‘time’, and ‘sex’; the two-way interactions ‘treatment by time’, ‘treatment by sex’, and ‘sex by time’; the three-way interaction ‘treatment by time by sex’ and a covariate all as fixed effects [24]. The pre-treatment value closest to dosing was used as the covariate.

If the interaction terms were significant (*P* ≤ 0.10 level for two-way interactions and *P* ≤ 0.05 for the three-way interaction), treated groups were compared to the control either within each sex (treatment by sex significant), within each time point (treatment by time significant) or main effect only (neither treatment by sex nor treatment by time significant).

French translation of the Abstract is available in Additional file 1.

## Results and discussion

### Dose administration

During the dosing period of the study, the mean doses of lotilaner administered for 1×, 3× and 5×, were 26.61, 79.26 and 131.24 mg/kg, respectively, for males, and 26.67, 79.28 and 131.30 mg/kg, respectively, for females. Thus, the target dose rate planned in the dosage group was achieved for all test article treated animals.

### General health, detailed clinical observations and ophthalmoscopic evaluations

No mortality was observed during the course of the study with the exception of one death of one female kitten (3× group: 78 mg/kg), which was found dead on study day 143. The animal had been anesthetized prior to electrocardiogram measurement; however, during monitoring at the time of removal for the procedure she was found dead in the cage. There were no macroscopic observations in this animal and microscopic findings were limited to mild unilateral tubular regeneration in the kidneys. Due to the focal unilateral distribution, low severity, and the presence of similar findings in multiple animals at the terminal necropsy including controls, this kidney finding was not considered to be related to test article administration and was considered not to be the primarily cause for this animal’s death during anesthesia. The animal’s death was considered most likely an aberrant response to anesthesia. Thus, there were no treatment-related adverse findings in general health observations. No clinical signs associated with lotilaner administration at the tested doses were noted during the study. Occasional findings observed, such as abdominal distension, soft/watery feces, mild prolapsed rectum, swollen muzzle, and scabbed areas at random sites, were considered incidental since they commonly occurred in animals of this strain and age, were present at a low incidence, were observed in control animals as well, and/or were not dose-dependent.

No treatment-related effects were observed during ophthalmoscopic examinations. One female at 78 mg/kg (3× group) had chorioretinitis in the right eye at the interim (study day 99) and terminal (study day 211) examinations. The finding was considered incidental since it was seen in only one mid-dose animal and was present in only one eye.

### Body weights and food consumption

Body weights of the males at 78 mg/kg (3×) were significantly lower (*t*_(27)_ = 2.69, *P* = 0.0122) than those of the control males, averaged over all time points. Since no effects were observed in males at the higher dose of 130 mg/kg (5×), dose-dependency to treatment could not be established. This correlates with the reduction in food consumption in this group of males. No effects on body weight were observed in females at any dose level. Food consumption was reduced in treated males (3×) compared to controls, but not in females. The changes were minor and considered non adverse. The reduction in food consumption only correlated to decreased body weight in males at 78 mg/kg (3×). Wet food consumption was reduced in males at 26 mg/kg (1×) level compared to controls. This was considered most likely incidental to treatment since it was not seen at the higher doses in males or in females at any dose level. Therefore, it was concluded that the test article had no significant effect on body weights or food consumption.

### Physical/neurological examinations

No clinically relevant abnormalities attributable to the treatment were observed during scheduled physical/neurological examinations.

### Electrocardiographic evaluations

No changes were recorded after auscultation in all treated groups. All electrocardiograms were qualitatively and quantitatively within normal limits with the exception of one animal treated at 130 mg/kg (5×) where an electrical alternans on the terminal ECG was recorded. Intraventricular conduction disturbances are not uncommon in cats [25]. Intraventricular conduction disturbances were not associated with clinical signs and hence the conduction disturbance is not considered adverse.

When absolute group mean values were evaluated statistically and compared to interval matched vehicle control values, the only significant differences involved the QRS duration (at days 199 (*t*_(69)_ = 1.72, *P* = 0.0892) and 224 (*t*_(69)_ = 2.22, *P* = 0.0299) at 1× and at day 59 at 5× (*t*_(70)_ = 1.81, *P* = 0.0748). The QRS duration was shorter in the 1× group at study day 199 and terminal intervals and longer in the 5× group at study day 59 interval (Table 2). As the magnitude of the differences was mild, the changes were in opposite directions and only occurred at one of the high dose intervals with no reproducibility, the differences are likely due to normal biological variability and not a test article effect.

**Table 2:** Comparisons (statistically significant) between control and treated groups for QRS duration

Parameter	Sex	Time	Control group	Treatment group	*t*-value	*P*-value
QRS Duration	Pooled	Day 199	0 mg/kg	1× (26 mg/kg)	*t*_(69)_ = 1.72	0.0892
		Terminal	0 mg/kg	1× (26 mg/kg)	*t*_(69)_ = 2.22	0.0299
		Day 59	0 mg/kg	5× (130 mg/kg)	*t*_(70)_ = 1.81	0.0748

### Clinical pathology

Statistically significant decreases in neutrophils were observed in males and females at multiple time points (study day 8 through 223; for males: up to *t*_(25)_ = 3.60, *P* = 0.0014; for females: up to *t*_(24)_ = 4.22, *P* = 0.0003) at all dose levels in comparison with control animals (Table 3). This finding was considered test article-related, based on its consistency over time and in both sexes, but considered not to have clinical relevance due to the absence of other correlating abnormalities in other parameters. There were statistically significant decreases in APTT in females overall (all time points combined) at 1× (*t*_(15)_ = 2.02, *P* = 0.0606) and 3× (*t*_*(*15)_ = 1.84, *P* = 0.0855), relative to controls. There were also significant decreases (up to *t*_(34)_ = 2.08, *P* = 0.0457) in prothrombin times at multiple time points (study day 36 through 223) at 1×, 3× and 5×. There were statistically significant increases in globulin in pooled (both sexes) on study day 56 at 3× (*t*_(26)_ = 1.88, *P* = 0.0709), and cholesterol in pooled samples at multiple time points (study day 36 through 223) at 3× and 5× (up to *t*_(17)_ = 4.11, *P* = 0.0008). These effects were minor, did not follow a consistent dose response, lacked correlation to pathology or other study endpoints and generally remained within expected historical ranges and hence considered not clinically relevant. There were no treatment-related changes in urinalysis parameters.

**Table 3:** Comparisons (statistically significant) between control and treated groups for clinical pathology

Parameter	Sex	Time	Control group	Treatment group	*t*-value (highest)	*P*-value (lowest)
Neutrophils	Male	Day 8-Day 223	0 mg/kg	1× (26 mg/kg)	*t*_(25)_ = 2.62	0.0147 (on day 223)
				3× (78 mg/kg)	*t*_(25)_ = 2.85	0.0087 (on day 120)
				5× (130 mg/kg)	*t*_(25)_ = 3.60	0.0014 (on day 223)
	Female	Day 8-Day 223	0 mg/kg	1× (26 mg/kg)	*t*_(26)_ = 2.74	0.0110 (on day 196)
				3× (78 mg/kg)	*t*_(24)_ = 4.22	0.0003 (on day 8)
				5× (130 mg/kg)	*t*_(26)_ = 3.67	0.0011 (on day 196)
APTT	Female	Overall	0 mg/kg	1× (26 mg/kg)	*t*_(15)_ = 2.02	0.0606
				3× (78 mg/kg)	*t*_(15)_ = 1.84	0.0855
Prothrombin time	Pooled	Day 36-Day 223	0 mg/kg	1× (26 mg/kg)	*t*_(34)_ = 2.08	0.0457 (on day 92)
				3× (78 mg/kg)	*t*_(37)_ = 2.04	0.0490 (on day 223)
				5× (130 mg/kg)	*t*_(34)_ = 1.70	0.0982 (on day 36)
Globulin	Pooled	Day 56	0 mg/kg	3× (78 mg/kg)	*t*_(26)_ = 1.88	0.0709
Cholesterol	Pooled	Day 36-Day 223		3× (78 mg/kg)	*t*_(14)_ = 3.42	0.0041 (on day 223)
				5× (130 mg/kg)	*t*_(17)_ = 4.11	0.0008 (on day 120)

### Organ weights and gross and microscopic examinations

There were no lotilaner-related macroscopic findings at terminal necropsy. Statistically significant absolute organ weight changes in the spleen, relative organ weight changes in adrenal and kidney, and both absolute and relative organ weight changes in brain and thyroids/parathyroids (relative) were considered incidental since there were no microscopic correlates or relevant clinical pathology changes. No definitive test article-related changes were observed microscopically.

### Pharmacokinetic analysis

**Fig. 1:**
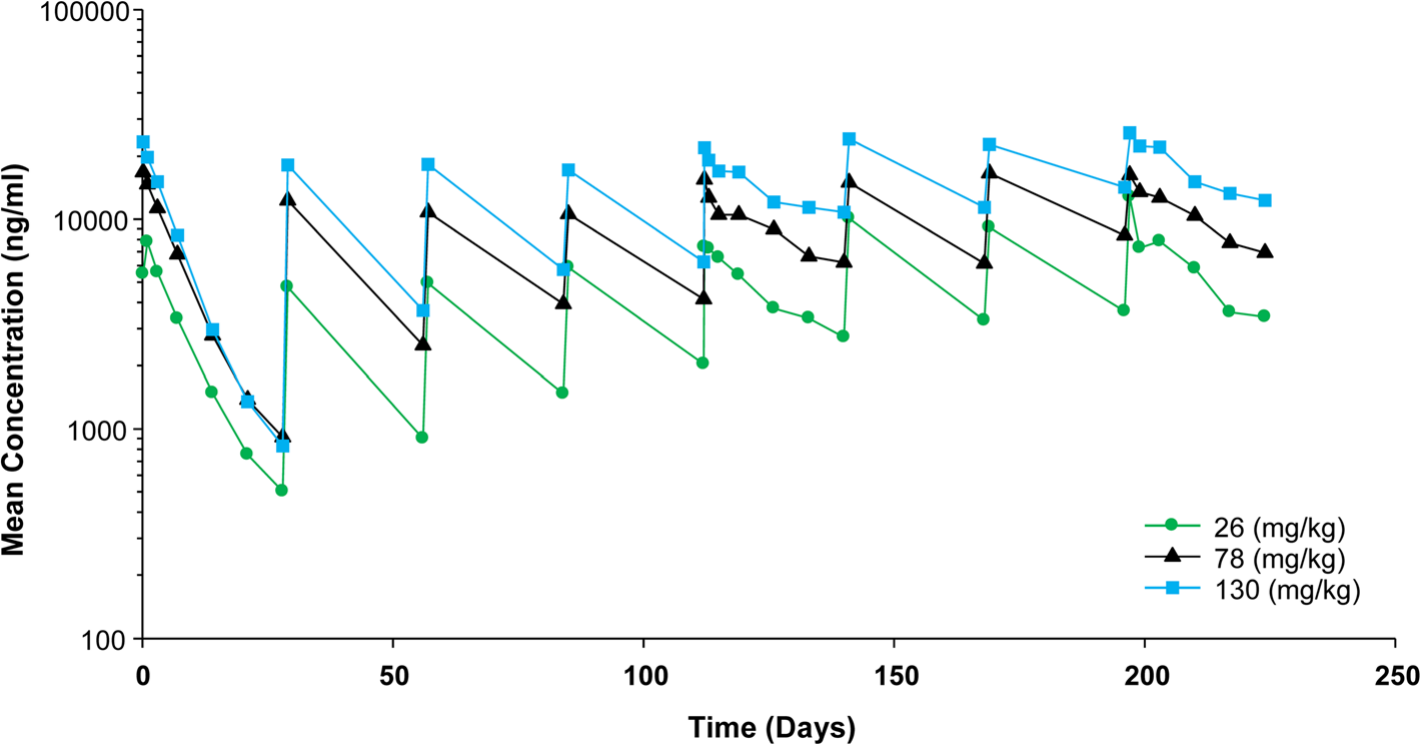
Mean lotilaner whole blood concentration-time profiles following eight consecutive monthly oral administrations of 26 (1×), 78 (3×), and 130 (5×) mg/kg

Regarding pharmacokinetics, lotilaner was not quantifiable (below LLOQ) in any untreated animals (control group). The variability of lotilaner C_max_ and AUC_0-672h_ was similar between animals and months throughout the study. This demonstrates consistent and adequate exposure of all treated cats. Mean systemic exposure (AUC_0-672h_) and C_max_ values of lotilaner increased with increasing dose in a less than dose proportional manner, especially in the 5× group, which was approximately 3-fold instead of 5-fold (Fig. 1). A moderate degree of accumulation (from single treatment to steady state (approximately 5 months) with an accumulation ratio of approximately 2) was observed for lotilaner. This was expected as a normal consequence of the relatively long half-life (approximately 4 weeks) of the product.

## Conclusions

Lotilaner, when administered once monthly over eight months at the highest recommended dose and overdoses of three and five-fold, to 8-week-old healthy kittens, is well tolerated. The results show that lotilaner flavoured chewable tablets have a wide safety margin when administered at monthly intervals to kittens and cats, male or female, at the highest dose band rate of 26 mg/kg.

## Additional file

Additional file 1: French translation of the Abstract. (PDF 41 kb)

## Abbreviations

ANOVA: Analysis of variance
APPT: Activated partial thromboplastin time
ARRIVE: Animal Research Reporting *in vitro* Experiments
FDA: Mean systemic exposure from 0 to 672 h
CFR: Code of Federal Regulations
Cmax: Peak blood concentration
ECG: Electrocardiograms
FDA: United States Food and Drug Administration
GLP: Good laboratory practice
OECD: Organization for Economic Cooperation and Development
RMANCOVA: Repeated measures analysis of covariance
VICH: Veterinary International Conference on Harmonization
