# Supplementary material for: Safety of lotilaner flavoured chewable tablets (CredelioTM) after oral administration in cats
Source: Parasit Vectors. 2018 Jul 13;11:409. doi: 10.1186/s13071-018-2969-3 (PMC6044082; doi:10.1186/s13071-018-2969-3)
Supplement: Supplementary file 1 — French translation of the Abstract. (PDF 41 kb) [file 13071_2018_2969_MOESM1_ESM.pdf]

Innocuité des comprimés aromatisés à croquer au lotilaner (Credelio^{TM}) après administration par voie orale à des chats

Emmanuelle A. Kuntz[1]* et Srinivas Kammanadiminti[2]

[1]Elanco Animal Health, Mattenstrasse 24a, CH-4058, Bâle, Suisse
[2]Elanco Animal Health, 2500 Innovation Way, Greenfield, IN 46140, États-Unis.

*Correspondance : emmanuelle.kuntz@elanco.com

Adresses électroniques :
Emmanuelle A. Kuntz : emmanuelle.kuntz@elanco.com
Srinivas Kammanadiminti : srinivas.kammanadiminti@elanco.com

Résumé

Contexte : Le lotilaner est un nouveau composé de la famille des isoxazolines, indiqué dans le traitement des infestations par les tiques et les puces chez le chat. Cette étude de laboratoire randomisée, réalisée en aveugle et en groupes parallèles, portait sur l'innocuité de comprimés à croquer aromatisés vanille et levure (Credelio^{TM}, Elanco) sur des chatons en bonne santé d'au moins 8 semaines. Des comprimés au lotilaner ont été administrés par voie orale, une fois par mois pendant huit mois à des doses correspondant à une, trois et cinq fois la limite supérieure de la dose maximale recommandée (26 mg/kg).

Méthodes : L'innocuité des comprimés aromatisés à croquer au lotilaner a été évaluée lors de leur administration par voie orale à des chatons en bonne santé, toutes les 4 semaines pendant 8 mois à la dose maximale recommandée, c'est-à-dire 1 × (26 mg/kg) et à des doses élevées, à savoir 3 × (78 mg/kg) et 5 × (130 mg/kg). Seize chatons mâles et seize chatons femelles âgés de huit semaines, en bonne santé, pesant en moyenne 0,79 kg et 0,75 kg, respectivement, ont été répartis aléatoirement entre un groupe témoin et un groupe traité avec du lotilaner à raison de 26 mg/kg (1×), 78 mg/kg (3×) ou 130 mg/kg (5×), toutes les 4 semaines pendant 8 mois. Les animaux du groupe témoin ont reçu des comprimés de placebo. Tous les animaux ont été nourris dans les 30 minutes précédant le traitement. L'évaluation de l'innocuité reposait sur l'observation : de l'état sanitaire global, d'observations cliniques détaillées et

d'examens physiques/neurologiques complets incluant examens ophtalmologiques, électrocardiogramme (ECG), analyses biologiques (hématologie, biochimie clinique et analyse d’urine), consommation d’eau et d’aliment, poids corporel, prélèvements sanguins destinés aux analyses pharmacocinétiques, ainsi que des examens macroscopiques et microscopiques des organes.

Résultats : L’exposition systémique au lotilaner a été confirmée au cours de l’étude pour tous les animaux traités, mais pas pour ceux du groupe témoin. Aucun effet lié au traitement n’a été observé lors des examens cliniques quotidiens, ni sur la consommation alimentaire (humide), les examens ophtalmoscopiques, cliniques/neurologiques ou microscopiques. Des différences significatives ont cependant été notées pour certains paramètres d'analyses biologiques, pour le poids corporel, la consommations alimentaire (sèche), les électrocardiogrammes et le poids des organes. Aucune des observations réalisées n’a cependant été considérée comme cliniquement significative.

Conclusions : Le lotilaner est bien toléré en administration mensuelle pendant 8 mois chez des chatons de 8 semaines en bonne santé, à la dose maximale recommandée et à des doses trois et cinq fois supérieures à celle-ci.

Mots clés : Lotilaner, Credelio™, innocuité, chat, voie orale
